# 

**Published:** 1894-02

**Authors:** 


### PENNSYLVANIA STATE VETERINARY MEDICAL ASSOCIATION.

The annual meeting of the Pennsylvania State Veterinary Medical Association will be held at the College of Physicians, Philadelphia, March 6th and 7th.

Other associations will please note the time and appoint delegates to meet us.

W. H. Ridge, V.M.D.,

Secretary.

Trevose, Pa.

